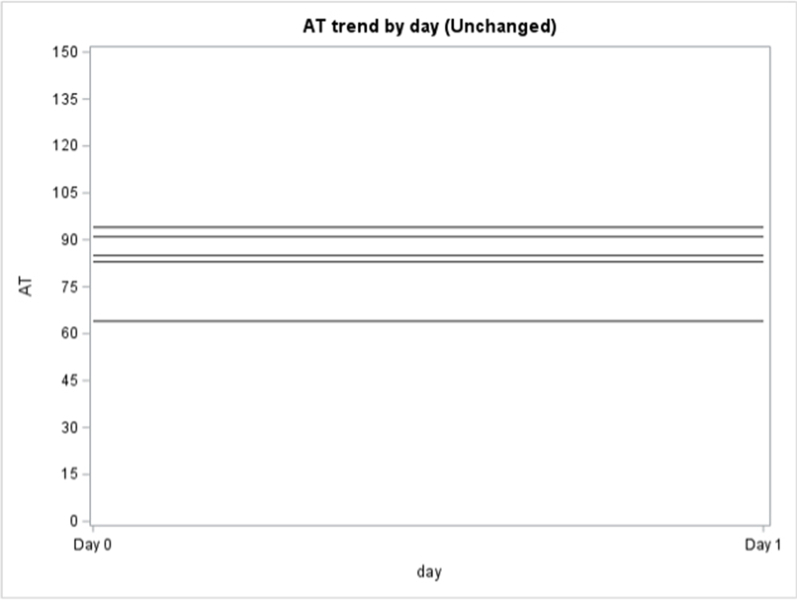# The Thromboembolism Heparinization and AntithrombiN Observational Study (THANOS-1)

**DOI:** 10.1016/j.rpth.2026.103367

**Published:** 2026-01-27

**Authors:** Christiana K. Prucnal, Grace Wang, Weixing Huang, Nora Horick, Ryan Mize, Isabel Dhar, Tyleah Brown, Sophie Flomenbaum, Kyle E. Chang, Timothy M. Matthews, Gregory A. Peters, Drew A. Birrenkott, Karsten Stannek, Eddie Eun Sang Lee, Sacha Uljon, Christopher Kabrhel

**Affiliations:** 1 Center for Vascular Emergencies, Department of Emergency Medicine, Massachusetts General Hospital, Harvard Medical School, Boston, Massachusetts, USA; 2 Department of Emergency Medicine, Harvard Medical School, Boston, Massachusetts, USA; 3 Department of Emergency Medicine, Massachusetts General Hospital, Boston, Massachusetts, USA; 4 Department of Emergency Medicine, Brigham and Women’s Hospital, Boston, Massachusetts, USA; 5 Department of Biostatistics, Massachusetts General Hospital, Harvard Medical School, Boston, Massachusetts, USA; 6 Department of Pathology, Hematology Laboratory, Massachusetts General Hospital, Harvard Medical School, Boston, Massachusetts, USA; 7 School of Medicine, California University of Science and Medicine, Colton, California, USA; 8 Faculty for Health, Witten/Herdecke University, Germany; 9 UCD School of Medicine, University College Dublin, Ireland

**Keywords:** antithrombins, heparin, pulmonary embolism, therapy, venous thromboembolism

## Abstract

**Background:**

Acute pulmonary embolism (PE) affects both hemodynamics and the clotting system, and changes in clotting protein activity may affect the effectiveness of anticoagulation. For example, PE may represent an acute, acquired antithrombin (AT)-deficient state, which may limit heparin effectiveness. However, the incidence and clinical effects of acquired AT deficiency after PE are not known.

**Objectives:**

Our primary aim was to calculate the proportion of patients with PE and acquired AT deficiency, defined a priori as <80% functional activity. We also analyzed <90%, <100%, and <110% AT activity. Secondary aims were to identify clinical factors and outcomes associated with acquired AT deficiency.

**Methods:**

We performed a prospective, observational study of patients diagnosed with acute PE without contraindications to heparin anticoagulation. We obtained blood within 24 hours after positive PE imaging and measured AT activity. Outcomes were culled from the medical record.

**Results:**

We analyzed 200 patients. Mean age was 62 ± 16 years, and 120 (60%) were men. Fifty-four (27%) patients had <80%, 111 (56%) <90%, and 159 (80%) <100% AT activity. Low AT activity (<80%) was associated with longer hospital length of stay (*P* < .0001), intensive care unit admission (*P* = .0085), and adverse clinical outcomes (*P* = .0042), but not subtherapeutic anticoagulation.

**Conclusion:**

Acquired AT deficiency is common after acute PE, occurring in at least one-quarter of all patients. Low AT levels are associated with adverse clinical outcomes, intensive care unit admission, and longer hospital length of stay, but whether this is related to subtherapeutic anticoagulation is not clear.

## Essentials

• The incidence of acquired antithrombin deficiency after pulmonary embolism (PE) is not known.
• We assessed antithrombin activity and outcomes in 200 patients with acute PE.
• Low antithrombin, present in 27% of patients, was associated with adverse clinical outcomes.
• Acquired antithrombin deficiency is common after PE and may affect treatment and outcomes.

## Graphical abstract

**Figure:**
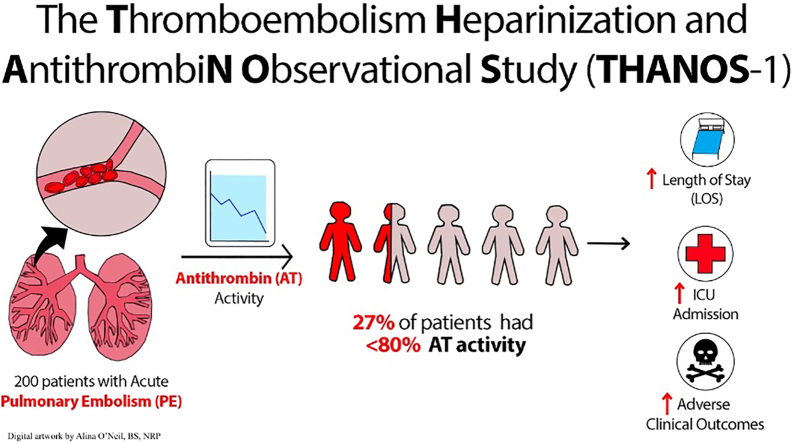

## 1 Introduction

Pulmonary embolism (PE) imposes a major disease burden globally and is an important source of morbidity and mortality in the United States [[1], [2], [3], [4]]. Acute PE affects both hemodynamics and the clotting system, and changes in clotting protein activity may reduce the effectiveness of anticoagulation.

Antithrombin (AT) is a potent inactivator of thrombin and factor (F)Xa and the major inhibitor of blood coagulation. Heparin binding to AT catalyses the inactivation of thrombin 1000-fold, thereby preventing fibrin formation and inhibiting thrombin-induced activation of platelets, FV and FVIII. Thus, physiological levels of AT are required for adequate anticoagulation with unfractionated heparin (UFH), but likely also for low-molecular-weight heparin (LMWH) and fondaparinux, which also work by binding with AT. Acquired AT deficiency is well described in other clinical conditions. In patients undergoing cardiac surgery, acquired AT deficiency is the cause of heparin resistance in 65%-85% of cases [5,6] and treatment with exogenous AT concentrate prolongs clotting time [5,7].

Little is known about the incidence and clinical effects of acquired AT deficiency after PE. Anecdotal evidence and prepublication data suggest that far more patients are AT deficient after an acute PE than would be expected due to genetic antithrombin deficiency [8,9]. Whether this is due to the clot burden of PE itself or some other mechanism is unclear and the actual incidence of acquired AT deficiency after PE is not known. Additionally, multiple studies have shown that many (40%) patients with PE do not achieve therapeutic anticoagulation in the first 48 hours of UFH therapy [10,11]. Delayed anticoagulation is associated with increased in-hospital and 30-day mortality after PE [12,13]. Acquired AT deficiency may contribute to these adverse clinical outcomes, possibly by decreasing the effectiveness of heparin. To address this knowledge gap, we performed an observational study of consecutive patients diagnosed with acute PE to quantify the proportion who are AT deficient, determine whether low AT activity is associated with adverse clinical outcomes, and evaluate whether low AT activity is associated with subtherapeutic heparinoid anticoagulation (UFH or LMWH).

## 2 Methods

The study was approved by the Mass General Brigham institutional review board.

### 2.1 Study design and setting

We performed a prospective, observational study at Massachusetts General Hospital (MGH), a large, urban, tertiary referral center with an annual emergency department (ED) census of >120,000 patients per year.

### 2.2 Screening and enrollment

Our goal was to enroll 200 consecutive adult patients diagnosed with radiographically confirmed PE. Trained research staff from the MGH Emergency Medicine Center for Vascular Emergencies (centerforvascularemergencies.org) screened and approached patients for study enrollment and obtained informed consent. We identified patients with PE using a combination of live screening in the ED and daily review of computed tomography pulmonary angiography (CTPA) and ventilation/perfusion (V/Q) scans performed on ED patients by the MGH Department of Radiology. Inclusion criteria included age at least 18 years at time of enrollment, imaging-confirmed acute PE diagnosed at MGH or transferred to MGH from an outside clinic or hospital, and a plan to treat with anticoagulation (according to the treating physician). Patients of all clinical PE severity were eligible. Exclusion criterion included age less than 18 years, inability to provide informed consent, use of therapeutic or prophylactic anticoagulation in the 24 hours prior to PE diagnosis, and unlikely survival for at least 24 hours after enrollment. Given our interest in the interaction between AT and heparin, we also excluded patients with a contraindication to heparin anticoagulation. However, patients who did not have a contraindication but were treated with a nonheparin anticoagulant at the discretion of their care team were eligible for inclusion.

We defined acute PE as a filling defect consistent with thromboembolism visualized in a pulmonary artery seen on CTPA or a high-probability V/Q scan. We based CTPA and V/Q findings on the clinical interpretations of board-certified radiologists not affiliated with the study. For the purposes of analysis, we defined a deep vein thrombosis (DVT) as a visualized clot (eg, an incompressible vein) seen on venous ultrasound, based on the clinical read of either a board-certified radiologist or a certified emergency sonographer performing a bedside ultrasound, not affiliated with the research study.

### 2.3 Data acquisition

We collected clinical and demographic data by performing interviews with study subjects and clinicians and by reviewing the electronic medical record (Epic). We collected the following: patient demographics (age, sex, race, ethnicity, and insurance status); comorbid Illnesses (prior venous thromboembolism [VTE], cardiac disease, pulmonary disease, cancer [solid tumor or hematologic], inflammatory/rheumatologic conditions, and hematologic conditions); characteristics of the PE (location of thrombus, presence of right heart dysfunction on echocardiogram or computed tomography); biomarkers (troponin, brain natriuretic peptides, and D-dimer); anticoagulation assays (activated partial thromboplastin time [APTT], anti-Xa, and AT); European Society of Cardiology risk classification (low, intermediate, and high); presence and location of residual DVT; clot in transit through the heart; PE therapies used (anticoagulation type and dose, thrombolysis, surgical or percutaneous thrombectomy, and extracorporeal membrane oxygenation); length of stay (LOS); disposition to intensive care unit (ICU); and death within 7 days of PE diagnosis. Race and ethnicity data were sourced from the electronic medical record. We entered data into a standardized data collection instrument using REDCap (www.projectredcap.org). Clinicians providing care to study participants were unaware of AT test results.

### 2.4 Blood sample collection

Serum testing for the study was drawn from a peripheral vein by phlebotomy-trained staff at 3 time points: (1) day 0 (within 24 hours of PE diagnosis, based on the time of the imaging study that confirmed acute PE); (2) day 1 (24-47 hours after diagnosis); and (3) day 2 (48-72 hours after diagnosis). Whenever possible, study blood draws were coordinated with clinical blood draws. Research laboratory tests, including and AT activity, APTT and anti-Xa levels, were performed at the time of the blood draw, rather than frozen for later testing.

### 2.5 Blood sample analysis

Venous blood was drawn into silicone-coated blue-top tubes (9 parts whole blood to 1 part 3.2% sodium citrate) and inverted 10 times to ensure proper mixing of blood and citrate. On receipt in the laboratory, samples were centrifuged at 4000 revolutions per minute for 10 minutes to achieve a platelet count of <10,000/μL and carefully decanted. Anti-Xa tests were performed on all specimens using Anti-Xa screen (Stachrom Heparin) and a STA-R Max analyzer (Diagnostica Stago, Inc) per manufacturer instructions. APTT tests (STA-PTT Automate 5; Diagnostica Stago; #00595) were also run on the STA-R Max analyzer. AT activity was measured with the STA-R Max analyzer using STA-Stachrom ATIII (REF 00596) kit according to manufacturer instructions.

### 2.6 Study endpoints

Our primary outcome was the proportion of subjects with acquired AT deficiency measured on a blood sample drawn <24 hours after a PE diagnosis (ie, day 0). However, there is no standard definition of acquired AT deficiency in PE. Therefore, we defined our primary outcome as <80% functional AT activity—the lower limit of normal AT activity used to diagnose hereditary AT deficiency [14]. Acknowledging that <80% may not be the appropriate cutoff or adequate to enable effective heparin anticoagulation in PE, we also performed several a priori sensitivity analyses in which we defined acquired AT deficiency as <90%, <100%, and <110% functional AT activity. While 80% AT activity is considered deficient for patients with genetic AT deficiency, there is no established threshold that defines low AT in the setting of acute PE requiring anticoagulation. Additionally, previous studies have shown that changes in AT activity can be associated with VTE in the setting of acute illness, even when AT activity is >90%. Therefore, we decided a priori to take an agnostic approach to the definition of low AT, allowing for the possibility that normal or even supranormal AT activity might be required to achieve therapeutic anticoagulation in the setting of an acute thromboembolism [15,16]. We performed univariate and multivariable analyses to identify factors associated with acquired AT deficiency.

### 2.7 Secondary outcomes

In addition to initial AT activity, we described AT activity trends over time (on days 0, 1, and 2). We also performed exploratory analyses to determine whether patients with large central PE (saddle, main pulmonary artery, or clot in transit) or PE with residual DVT were more likely to have acquired AT deficiency. We also evaluated whether acquired AT deficiency was associated with more complex hospitalization, reflected by longer hospital lengths of stay or admission to an ICU. Additionally, we assessed whether patients with acquired AT deficiency were more likely to have an adverse clinical outcome, which we, a priori, defined as a composite of death within 7 days, hemodynamic instability, need for thrombolysis, thrombectomy (open surgical or catheter-based), need for extracorporeal membrane oxygenation, or admission to the ICU.

Lastly, in the subpopulation of patients initially treated with at least 1 dose of UFH or LMWH, we explored whether acquired AT deficiency was associated with (sub)therapeutic heparin anticoagulation. We used initial AT activity (on day 0) for this analysis to minimize potential changes in AT activity associated with prolonged heparin exposure. AT activity was dichotomized according to each a priori definition of acquired AT deficiency (ie, <80%, <90%, <100%, and <110% AT activity). We defined (sub)therapeutic heparin anticoagulation based on a combination of APTT and anti-Xa levels for patients treated with UFH, and anti-Xa levels for patients treated with LMWH. We examined this relationship 3 ways. First, we compared initial AT activity to the first APTT value that was drawn at least 4 hours after initiation of UFH. If a patient on UFH did not have an APTT, we used the anti-Xa level provided it was drawn 4 hours after the initiation of UFH. Patients on UFH were considered subtherapeutic if their first APTT was <70 seconds or their first anti-Xa was <0.3 IU/mL. Patients on LMWH were considered subtherapeutic if their first anti-Xa drawn at least 4 hours after the initial dose was <0.5 IU/mL. These targets align with values described in the literature and our institutional guidelines, although we recognize that variation can exist between institutions [[17], [18], [19]]. Second, we compared initial AT activity with the percentage of time a patient was therapeutic on UFH or LMWH during the first 72 hours of anticoagulation. We defined each patient’s status as therapeutic (or subtherapeutic) using the APTT and anti-Xa cutoffs as described earlier [[17], [18], [19]]. We then calculated the percentage of time each patient was in therapeutic (or subtherapeutic) range. We censored patients at hospital discharge, start of an anticoagulant other than UFH or LMWH, treatment with an advanced intervention (eg, systemic thrombolysis), death, or completion of study follow up (72 hours). When patients changed from receiving UFH to LMWH or vice versa, no person time was contributed until the next appropriately timed anti-Xa or APTT resulted. We conducted a sensitivity analysis where we alternatively carried forward the preceding therapeutic (or subtherapeutic) status when patients changed from UFH to LMWH (or vice versa). Finally, we evaluated whether time to achieve therapeutic status varied significantly by initial AT activity.

### 2.8 Statistical analyses

Data were exported to SAS v 9.4 (SAS Institute) for analysis. Simple proportions were used to quantify acquired AT deficiency at <80%, <90%, <100%, and <110% activity. We performed univariate and multivariable logistic regression to identify clinical characteristics associated with acquired AT deficiency. Clinical covariates with a *P* value of <.2 on univariate analysis were included in multivariable logistic models. We evaluated the associations between acquired AT deficiency, adverse clinical outcomes, and (sub)therapeutic anticoagulation using the chi-squared tests. We performed a survival analysis looking at time to therapeutic status with exposure being AT level, both as a continuous variable and as the predefined categories of acquired AT deficiency (AT < 80 vs AT ≥ 80, AT < 90 vs AT ≥ 90, AT < 100 vs AT ≥ 100, AT < 110 vs AT ≥ 110). The significance level for statistical tests was set at 0.05 without adjustment for multiple comparisons due to the exploratory nature of the analyses.

## 3 Results

**Supplementary Figure 1:**
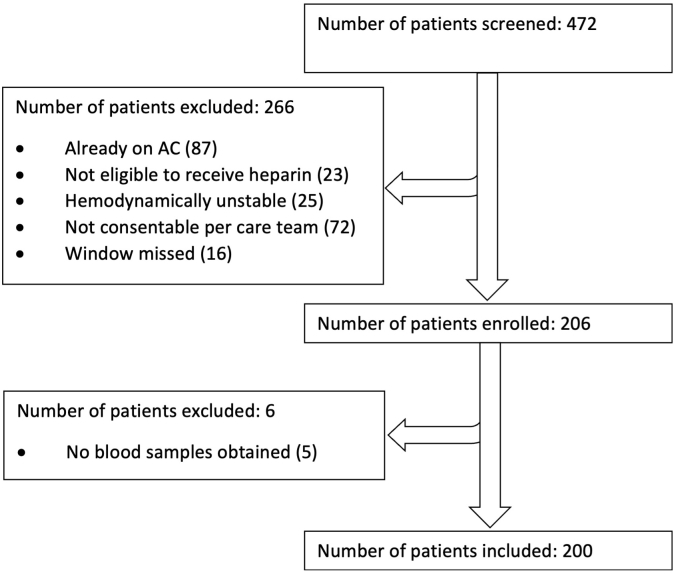


We screened 472 patients and enrolled 206 (Supplementary Figure 1). Six patients who were not treated with anticoagulation or in whom we were unable to obtain a blood sample were excluded. The remaining 200 patients had an average age of 62 ± 16 years, 120 (60%) were men, 158 (79%) were of White race, 20 (10%) were of Black race, and 18 (9%) were of Hispanic ethnicity (Table 1). The most common comorbid conditions included prior PE or DVT, malignancy, and smoking. More than a third of patients had central PE located in a saddle distribution, the main pulmonary artery, or intracardiac clot in transit (*n* = 82, 41%), 90 (53%) had concurrent DVT, and 56 (28%) had evidence of right heart strain on echocardiogram. Median hospital LOS was 100.5 hours with an IQR of 129.3 hours. ICU admission occurred in 38 (19%) patients. There were 47 patients who received only intravenous heparin and 83 patients who received only LMWH, as well as 54 patients who received both agents. There were 97 patients who received a direct oral anticoagulant (DOAC) and 92 patients who received a combination of intravenous heparin and/or LMWH as well as a DOAC during the study period. There were 14 patients (14%) who, following enrollment and blood collection, received an advanced treatment like thrombolysis or catheter-directed therapy.

**Table 1:** Characteristics of enrolled patients.

Variable	Patients (*N* = 200)
Age (y)	
Mean (SD)^a^	61.69 (16.1)
Median (IQR)^b^	62.76 (21.7)
Sex	
Female	80 (40.0)
Male	120 (60.0)
Race/ethnicity	
American Indian or Alaska Native	1 (0.5)
Asian	3 (1.5)
Black or African American	20 (10.0)
Hispanic or Latino Ethnicity	18 (9.0)
Native Hawaiian or Other Pacific Islander	0 (0.0)
White	158 (79.0)
Not reported, other, unknown	0 (0.0)
Body mass index	
Mean (SD)	29.9 (7.9)
Median (IQR)	28.1 (10.2)
Comorbidities	
Coronary artery disease	26 (13.0)
Atrial fibrillation/flutter	7 (3.5)
CHF (EF < 45%)	11 (5.5)
Stroke	11 (5.5)
Asthma/COPD	33 (16.5)
Smoker	42 (21.0)
DIC	0 (0.0)
Diabetes	35 (17.5)
Active malignancy (in treatment, palliation, or recently diagnosed)^c^	45 (22.5)
Inactive/past malignancy	43 (21.5)
Nephrotic syndrome	5 (2.5)
Renal insufficiency (eGFR <30 mL/min/1.73 m^2^)	4 (2.0)
Liver Disease^d^	13 (6.5)
Personal history of DVT/PE	49 (24.5)
Family history of DVT/PE	12 (6.0)
Hereditary/acquired thrombophilia^e^	5 (2.5)
Pregnancy or postpartum (within <4 wk)	0 (0.0)
Recent hospitalizations (within <4 wk)	32 (16.0)
Recent surgery (within <4 wk)	19 (9.5)
COVID-19 diagnosis (requiring hospitalization within <4 wk)	4 (2.0)
Recent burn (requiring hospitalization within <4 wk)	0 (0.0)
Central^a^ PE	82 (41)
DVT location	
Any location (proximal or distal)	90 (45.0-52.6)
Proximal	65 (32.5-37.8)^b^
Right heart strain	
Any RVD, RVH, septal flattening, or bowing on echocardiogram	56 (28.0)
Right heart strain on CTPA	72 (38.5)
RVSP, mean (SD) (*n* = 83)	43.8 (15.0)

Values are *n* (%) unless specified.

COPD, chronic obstructive pulmonary disease; CTPA, computed tomography pulmonary angiography; DIC, disseminated intravascular coagulation; DVT, deep vein thrombosis; EF, ejection fraction; PE, pulmonary embolism; RVD, right ventricular dilation; RVH, right ventricular hypokinesis; RVSP, right ventricular systolic pressure.

a Central = saddle, main arteries, or clot in transit.

b Percentage among patients who had imaging (eg, venous ultrasound) for DVT. There were 28 patients with unknown DVT-positive/negative status who were categorized as missing. We did not assume missing as negative.

c Types of active malignancy included as follows: prostate cancer (2), colon cancer (1), ovarian cancer (1), breast cancer (3), squamous cell cancer (5), multiple myeloma (2), gastric cancer (1), melanoma (1), pancreatic cancer (6), hepatocellular carcinoma (2), leukemia (1), glioblastoma (3), cholangiocarcinoma (3), unknown (2), duodenal adenocarcinoma (1), lung cancer (1), cutaneous T cell lymphoma (1), urothelial carcinoma (2), esophageal cancer (1), and multiple malignancies (prostate cancer and renal cell carcinoma; mantel cell lymphoma, prostate cancer, and squamous cell carcinoma; prostate cancer and colon cancer; endometrial cancer and breast cancer; colon cancer, cholangiocarcinoma, and urothelial carcinoma).

d Types of liver disease included as follows: alcoholic hepatitis (1), hepatic steatosis (7), cirrhosis (4), and cholangiocarcinoma (1).

e Types of thrombophilia included as follows: factor V Leiden (3), protein S deficiency (1), and other (1).

Fifty-four (27%) of 200 patients with acute PE had <80% AT activity (Table 2), and most patients with acute PE had <90% AT activity (Table 2). Among those with AT activity <80%, 21 (26%) had a central PE (defined as saddle, main pulmonary artery, or intracardiac clot in transit”), nd 33 (36%) had concurrent DVT.

**Table 2:** Clinical covariates by antithrombin (AT) activity after acute PE diagnosis.

Covariate	Day 1			
	AT <110%, *n* (%)	AT <100%, *n* (%)	AT <90%, *n* (%)	AT <80%, *n* (%)
All patients (*N* = 200)	164 (82)	159 (79.5)	111 (55.5)	54 (27)
Gender				
Male (*n* = 120)	104 (86.7)	101 (84.2)	70 (58.3)	35 (23.8)
Female (*n* = 80)	60 (75)	58 (72.5)	41 (51.3)	19 (29.2)
Age (y)				
>62 (*n* = 105)	86 (81.9)	83 (79.1)	58 (55.2)	30 (28.6)
≤62 (*n* = 95)	78 (82.1)	76 (80)	53 (55.8)	24 (25.3)
Race/ethnicity				
White (*n* = 158)	127 (80.4)	123 (77.9)	84 (53.2)	40 (25.3)
Black/African American (*n* = 20)	18 (90)	17 (85)	11 (55)	5 (25)
Hispanic (*n* = 18)	16 (88.9)	16 (88.9)	13 (72.2)	7 (38.9)
Asian/American Indian/Alaskan Native (*n* = 4)	3 (75)	3 (75)	3 (75)	2 (50)
Medical history				
Personal history of PE/DVT (*n* = 49)	40 (81.6)	40 (81.6)	26 (53.1)	12 (24.5)
Active malignancy (*n* = 45)	38 (84.4)	35 (78.8)	25 (55.6)	18 (40)
VTE diagnosis				
Central PE (*n* = 82)	70 (85.4)	67 (81.7)	43 (52.4)	21 (25.6)
DVT+ (*n* = 91^a^)	79 (86.8)	76 (83.5)	51 (56)	33 (36.3)

AT, antithrombin; DVT, deep vein thrombosis; PE, pulmonary embolism.

a There were 28 patients with unknown DVT-positive/negative status who were categorized as missing.

Among the 130 of 200 (65%) patients for whom AT activity was measured on days 0, 1, and 2, 62 (48%) had a mixed trend, meaning that AT activity decreased then increased (or vice versa) over time, 58 (45%) had consistently decreasing AT activity, and 10 (8%) had consistently increasing AT activity over the 3 days (Supplementary Figure 2). In the subpopulation of patients who were only treated with UFH or LMWH and had >1 AT level measured (*n* = 138), 79 (57%) had a decreasing trend in AT activity, 40 (29%) had a mixed trend, 14 (10%) had an increasing trend, and 5 (4%) stayed constant (Supplementary Figure 3).

Univariate analyses of patient factors associated with acquired AT deficiency, at each cutoff, are provided in Supplementary Table 1. Clinical covariates with a *P* value of <.2 on univariate analysis were included in multivariable logistic models. Table 3 shows the results of our multivariate analyses. A history of liver disease was significantly positively associated with AT activity of <80% (odds ratio [OR], 7.51; 95% CI, 1.67-33.83; *P* = .009) and <90% (OR, 4.89; 95% CI, 1.01-23.70; *P* = .049). A family history of DVT/PE (OR, 0.22; 95% CI, 0.05-0.92; *P* = .038) and active COVID-19 infection were significantly negatively associated with AT activity <110% (OR, 0.07; 95% CI, 0.00-0.98; *P* = .049), although the clinical relevance of an activity level above normal is not known. Other patient factors were not significantly associated with acquired AT deficiency.

**Table 3:** Multivariate analyses of clinical factors associated with low AT activity.

Factor	Covariate	Odds ratio (95% CI)	*P*
AT <80 vs ≥80	CHF (LVEF < 45%)	2.20 (0.52-9.38)	.28
	Stroke	0.18 (0.02-2.12)	.17
	Active malignancy	2.09 (0.79-5.54)	.13
	Nephrotic syndrome	1.41 (0.17-11.99)	.75
	Renal insufficiency	10.62 (0.41-276.7)	.15
	Liver disease	7.51 (1.67-33.83)	.008
	DVT positive	2.23 (0.95-5.22)	.064
	RHS on CTPA	1.73 (0.73-4.12)	.216
AT <90 vs ≥90	CHF (LVEF < 45%)	3.57 (0.71-17.86)	.12
	Asthma/COPD	2.33 (0.98-5.54)	.056
	Smoker	1.74 (0.80-3.80)	.16
	Liver disease	4.89 (1.01-23.70)	.048
	RHS on echocardiogram	1.81 (0.91-3.57)	.089
	Black vs White race	1.34 (0.50-3.61)	.56
	Hispanic vs White race	2.01 (0.65-6.23)	.22
	Other vs White race	4.01 (0.39-41.23)	.24
	BMI > 28.1 kg/m^2^	0.75 (0.41-1.39)	.36
AT <100 vs ≥100	Smoker	3.08 (0.98-9.65)	.054
	COVID-19^a^	0.14 (0.02-1.29)	.083
	Gender (male vs female)	1.97 (0.97-4.00)	.061
AT <110 vs ≥110	Smoker	3.43 (0.66-17.79)	.14
	Family history of DVT/PE	0.22 (0.05-0.92)	.038
	COVID-19^a^	0.07 (0.00-0.98)	.049
	Gender (male vs female)	1.95 (0.80-4.77)	.14
	DVT positive	1.44 (0.58-3.57)	.42
	RHS on CTPA	2.10 (0.80-5.50)	.13

AT, antithrombin; BMI, body mass index; CHF, congestive heart failure; COPD, chronic obstructive pulmonary disease; CTPA, computed tomography pulmonary angiography; DVT, deep vein thrombosis; LVEF, left ventricular ejection fraction; PE, pulmonary embolism; RHS, right heart strain.

a COVID-19 refers to a positive test for SARS-CoV-2 infection (COVID-19) at the time of pulmonary embolism diagnosis.

On univariate analysis, acquired AT deficiency was associated with more complex hospitalizations, reflected in longer LOS or ICU admission (*P* <.2 at all AT activity cutoffs on univariate analysis), as shown in Table 4. To address potential confounding between patient factors (eg, age and comorbid illness) and the complexity of hospitalization, we separately included LOS and ICU admission in the multivariate model shown in Table 3. ICU admission remained significantly associated with low AT <90% (OR, 2.42; 95% CI, 1.01-5.59; *P* = .048) (Supplementary Table 2). Hospital LOS remained significantly associated with low AT <80% (OR, 1.01; 95% CI, 1.00-1.01; *P* < .001) and <90% (OR, 1.00; 95% CI, 1.00-1.01; *P* = .002) (Supplementary Table 3).

**Table 4:** Univariate analyses of hospital-based factors associated with low AT activity.

Hospital covariates (compare ±, or as stated)	AT <80 vs ≥80		AT <90 vs ≥90		AT <100 vs ≥100		AT <110 vs ≥110	
	Odds ratio (95% CI)	*P*	Odds ratio (95% CI)	*P*	Odds ratio (95% CI)	*P*	Odds ratio (95% CI)	*P*
ICU admission	2.69 (1.29-5.63)	.009	2.69 (1.21-5.84)	.015	2.47 (0.82-7.42)	.11	2.92 (0.84-10.09)	.091
Length of stay (h)	1.01 (1.00-1.01)	<.001	1.005 (1.00-1.01)	<.001	1.003 (1.00-1.01)	.067	1.004 (1.00-1.01)	.052

AT, antithrombin; ICU, intensive care unit.

Patients with acquired AT deficiency were significantly more likely to have an adverse clinical outcome at all low AT level definitions (Table 5). For example, 33 (30%) patients with AT <90% experienced an adverse outcome (OR, 3.34; 95% CI, 1.54-7.24; *P* = .002), while only 10 (11%) patients with AT ≥90% did. However, this was mostly driven by ICU admission. When we removed ICU admission from the composite definition, the association between low AT levels was no longer significant, likely due to loss of statistical power.

**Table 5:** Association between adverse clinical outcomes and AT activity.

AT activity	Adverse clinical outcome^a^		
	No (*n* = 157)	Yes (*n* = 43)	Total (*n* = 200)
AT < 80%			
No	122 (83.6)	24 (16.4)	146
Yes	35 (64.8)	19 (35.2)	54
		*P* = .0042; OR, 2.76 (95% CI, 1.36-5.61)	
AT < 90%			
No	79 (88.8)	10 (11.2)	89
Yes	78 (70.3)	33 (29.7)	111
		*P* = .0016; OR, 3.34 (95% CI, 1.54-7.24)	
AT < 100%			
No	37 (90.2)	4 (9.8)	41
Yes	120 (75.5)	39 (24.5)	159
		*P* = .0401; OR, 3.00 (95% CI, 1.01-8.97)	
AT < 110%			
No	33 (91.7)	3 (8.3)	36
Yes	124 (75.6)	40 (24.4)	164
		*P* = .0337; OR, 3.55 (95% CI, 1.03-12.19)	

Values are *n* (%) unless specified.

AT, antithrombin; OR, odds ratio.

a Adverse clinical outcome was defined as any of the following: death within 7 d, hemodynamic collapse, thrombolysis, thrombectomy, extracorporeal membrane oxygenation, or intensive care unit admission.

We did not find a consistent association between acquired AT deficiency at time of PE diagnosis and (sub)therapeutic heparin anticoagulation. We found no statistically significant association between acquired AT deficiency and initial response to heparin anticoagulation (Table 6). Comparing acquired AT deficiency to the percentage of time a patient was therapeutic on UFH or LMWH during the first 72 hours of anticoagulation, patients with <90% AT activity were more likely to be therapeutic than patients with ≥90% AT activity. On average, patients with AT <90%, were therapeutic 7.5 hours (95% CI, 1.2-13.8) longer than patients with ≥90% activity (*P* = .021). Findings were similar whether we included or excluded patient time following a change from UFH to LMWH (or vice versa). At the other AT activity cutoffs (<80%, <100%, and <110%), we found no statistically significant association with (sub)therapeutic anticoagulation, nor did we find an association between AT activity and time therapeutic anticoagulation (Supplementary Table 4).

**Table 6:** Association between subtherapeutic heparinoid anticoagulation and AT activity.

AT activity	Subtherapeutic anticoagulation^a^		
	No (*n* = 111)	Yes (*n* = 65)	Total (*n* = 176)
AT < 80%			
No	75 (67.6)	49 (75.4)	124
Yes	36 (32.4)	16 (24.6)	52
		*P* = .2726	
AT < 90%			
No	46 (41.4)	26 (40.0)	72
Yes	65 (58.6)	39 (60.0)	104
		*P* = .8511	
AT < 100%			
No	23 (20.7)	9 (13.9)	32
Yes	88 (79.3)	56 (86.2)	144
		*P* = .2538	
AT < 110%			
No	19 (17.1)	8 (12.3)	27
Yes	92 (82.9)	57 (87.7)	149
		*P* = .3928	

Values are *n* (%) unless specified.

APTT, activated partial thromboplastin time; AT, antithrombin; LMWH, low-molecular-weight heparin.

a Subtherapeutic anticoagulation was defined as APTT < 70 or anti-Xa < 0.3 for intravenous heparin, or anti-Xa < 0.5 for LMWH.

## 4 Discussion

Ours is the first study to demonstrate that a large proportion of patients with acute PE have acquired AT deficiency. In fact, most patients with acute PE have <90% AT activity. This proportion is markedly greater than would be expected due to hereditary AT deficiency secondary to antithrombin mutation, the incidence of which is estimated at 2% among patients with history of VTE and between 1:500 and 1:5000 in the general population [9,20]. It is a limitation that we do not know for sure which, if any, patients in our study had genetic AT deficiency rather than acquired AT deficiency. However, 27% of patients in our study had <80% AT activity, a proportion significantly greater than could be explained by genetic deficiency, suggesting that nearly all of our cases are acquired AT deficiency. Whether this is due to consumptive physiology in acute PE, a milieu that predisposes to both low AT and VTE, or another etiology remains unclear. This finding raises questions regarding the treatment of PE, such as whether AT activity should be assessed prior to anticoagulant selection (eg, heparinoid vs other), whether AT repletion may improve anticoagulation in select patients, or whether measuring AT activity levels could be helpful in PE risk stratification. These questions require further study. While we did find a history of liver disease to be significantly associated with AT deficiency, this is a known association with impaired hepatic synthesis of the factor, and only a limited number of patients from the cohort had this comorbidity (13 patients).

We also found that PE patients with acquired AT deficiency had longer hospital LOS, were more likely to require ICU admission, and more likely to experience adverse clinical outcome than patients with normal AT activity. The reason for more complex hospitalizations among patients with low AT activity is not yet known, but treating clinicians were blind to the laboratory tests performed for our study, and AT activity is not commonly measured clinically, so it is unlikely that AT activity affected clinical decision making or biased our findings. Patients with acquired AT deficiency may be more clinically complex, have more severe PE, or are more challenging to anticoagulate in ways we did not measure. Following our study, it remains an open question whether low AT levels themselves cause or represent a marker of worse clinical outcomes. Both might be true. AT decreases in cases of liver failure, kidney disease, and in consumptive coagulopathies, all of which have negative consequences in terms of hospital stay and mortality. Conversely, the physiological functions of AT are related to coagulation and inflammation, so AT may be an independent predictor of clinical illness severity. Our study, therefore, should serve as an initial point for future investigations of the role AT plays in outcomes after PE.

Around half of the patients in our study had decreasing AT activity over time, although many patients had mixed trends over time, and some had rising AT activity. Because anticoagulation is typically started quickly after diagnosis, and we enrolled patients up to 24 hours after PE diagnosis, our study blood samples were frequently drawn after initiation of anticoagulation (92.5%). It is, therefore, possible that decreasing AT activity was related by prolonged exposure to heparin. However, previous studies have shown that AT levels are not affected by an initial bolus of UFH, so this should not have affected our initial AT activity results [21]. Another study of trauma patients also found decreasing trends in AT activity associated with prophylactic heparinoid anticoagulation, but in this study, low AT activity was associated with an increased (not decreased) risk of VTE, suggesting a complex relationship between AT activity and effective heparinoid anticoagulation [15].

Even with the availability of DOACs, UFH remains common in the treatment in acute PE [22]. This is despite the fact that many patients with PE treated do not achieve a therapeutic APTT within the first 24 hours of UFH anticoagulation [10]. Our current results reflect this, as a large proportion of our patients were initially treated with UFH, yet only 32 of 69 (46%) of those treated exclusively with UFH over the first 24 hours were always in or above the therapeutic range. Concerningly, 12 of 69 (17%) were always subtherapeutic. Because delayed treatment is linked to increased mortality after PE, it remains important that we understand the best way to manage heparin anticoagulation [12,13]. AT is the intrinsic glycoprotein by which heparins exert their therapeutic effect, so low AT activity level has been proposed as one possible explanation of the variation in response to heparin. However, we did not find a consistent pattern between acquired AT deficiency and subtherapeutic response to heparin anticoagulation. One possibility is that only very low levels of AT (eg, <60%) are associated with decreased response to heparin. Because the proportion of patients with very low AT levels is small, larger studies will be required to assess this. Interestingly, we found an isolated statistically significant association between AT level <90% and greater duration of therapeutic status on heparinoid treatment. This result is counterintuitive and does not replicate patterns seen in previous studies [5,7,15]. One possible explanation for these findings is that more aggressive heparin dosing not only lowers AT activity but also causes more time in therapeutic range. However, we think this is unlikely as we defined acquired AT deficiency based on levels drawn on day 0, and this mechanism would not explain an association being limited to the <90% AT activity group. Type I error is a possibility, and additional investigation is needed. It is also possible our definitions of (sub)therapeutic heparin anticoagulation were inadequate. We believe the association between low AT and therapeutic anticoagulation requires further study.

Other potential limitations of this study include its single site design, which may limit generalizability as well as its modest sample size, which may have rendered multivariate analyses underpowered to detect significant associations between acquired AT deficiency and markers of illness acuity or (sub)therapeutic anticoagulation. Additionally, this study was designed as an exploratory analysis without adjustments for multiple comparisons. As this is the first study to quantify AT levels at the time of acute PE diagnosis, future studies should validate our findings. We recognize that analytic methods can cause differences in anti-Xa levels. In our study, we primarily used APTT as our measure of therapeutic anticoagulation, partly due to this potential effect on anti-Xa, but future studies should consider analyzing anti-Xa results according to the reagent used and reanalyze the anti-Xa results separately.

## 5 Conclusions

Acquired AT deficiency is common after acute PE, occurring in at least one-quarter of all patients. Low AT levels are associated with adverse clinical outcomes, ICU admission, and longer hospital LOS, but whether this is related to subtherapeutic anticoagulation is not clear.

## Figures and Tables

**Supplementary Figure 2a:**
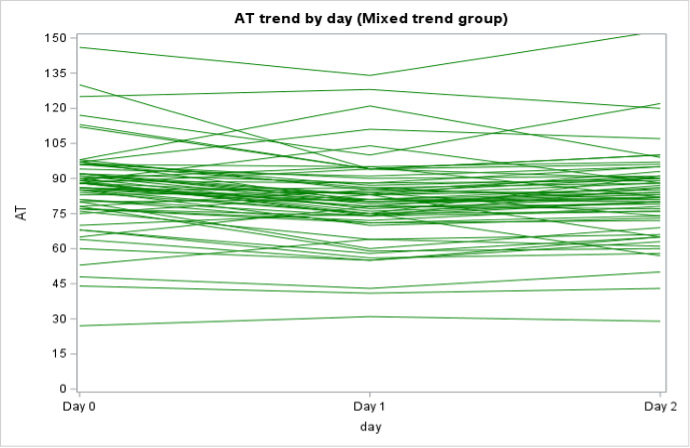


**Supplementary Figure 2b:**
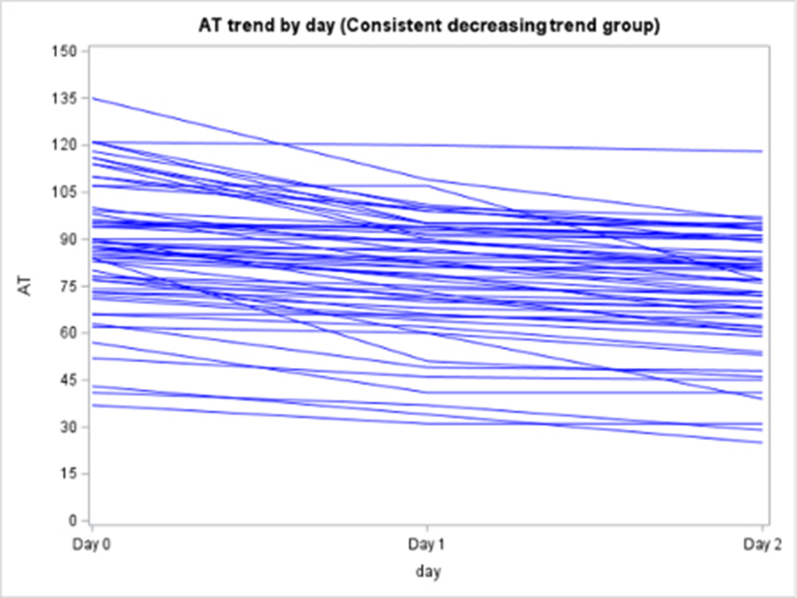


**Supplementary Figure 2c:**
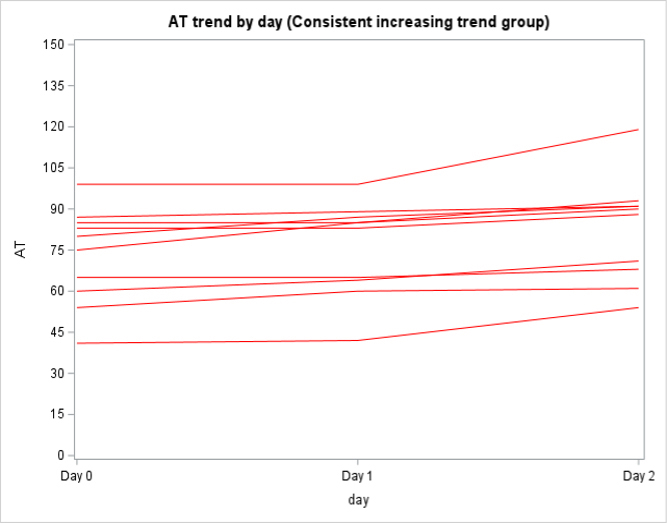


**Supplementary Figure 3a:**
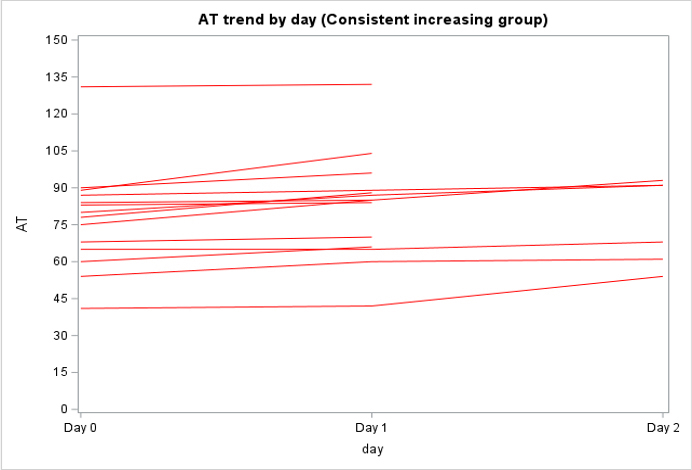


**Supplementary Figure 3b:**
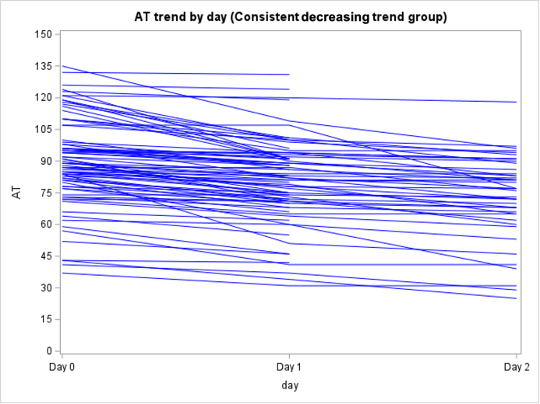


**Supplementary Figure 3c:**
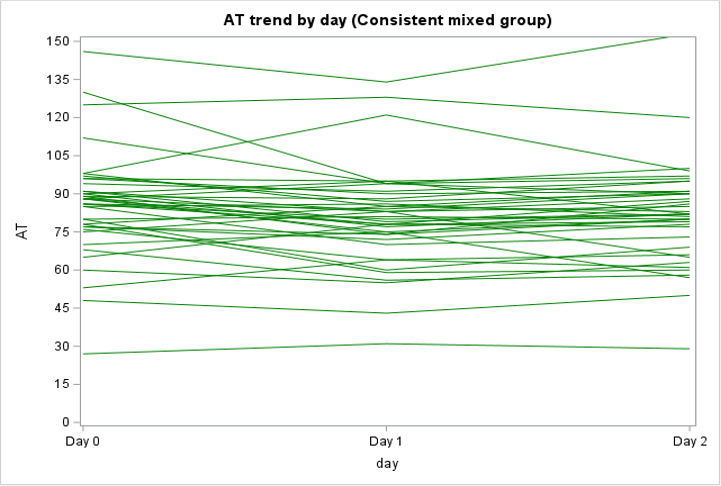


**Supplementary Figure 3d:**