# Supplementary material for: The Thromboembolism Heparinization and AntithrombiN Observational Study (THANOS-1)
Source: Res Pract Thromb Haemost. 2026 Jan 27;10(1):103367. doi: 10.1016/j.rpth.2026.103367 (PMC12934317; doi:10.1016/j.rpth.2026.103367)
Supplement: Supplementary Table 1 [file mmc1.docx]

**Supplemental Table 1:**  Univariate analyses of clinical factors associated with AT activity

|  | **AT <80 v. >=80** | | **AT <90 v. >=90** | | **AT <100 v. >=100** | | **AT <110 v. >=110** | |
| --- | --- | --- | --- | --- | --- | --- | --- | --- |
| **Clinical covariates**  (Compare +/-, or as stated) | Odds Ratio (95% CI) | p-value | Odds Ratio (95% CI) | p-value | Odds Ratio (95% CI) | p-value | Odds Ratio (95% CI) | p-value |
| Coronary Artery Disease | 0.61  (0.22, 1.70) | 0.3426 | 1.108  (0.48, 2.56) | 0.8095 | 1.485  (0.48, 4.58) | 0.4912 | 1.794  (0.51, 6.34) | 0.3637 |
| Atrial Fibrillation/Flutter | 1.09  (0.20, 5.76) | 0.9241 | 1.071  (0.23, 4.92) | 0.9293 | 0.633  (0.12, 3.38) | 0.5926 | 0.535  (0.10, 2.87) | 0.4653 |
| CHF (EF < 45%) | 2.38  (0.70, 8.15) | 0.1669 | 3.838  (0.81, 18.2) | 0.0908 | 2.684  (0.33, 21.6) | 0.3533 | 2.273  (0.28, 18.3) | 0.4410 |
| Stroke | 0.26  (0.03, 2.05) | 0.1999 | 1.430  (0.41, 5.05) | 0.5782 | 2.684  (0.33, 21.6) | 0.3533 | NC^A^  NC | 0.9787 |
| Asthma/COPD | 1.44  (0.65, 3.22) | 0.3714 | 2.452  (1.08, 5.60) | 0.0329 | 1.193  (0.46, 3.12) | 0.7184 | 0.985  (0.37, 2.60) | 0.9762 |
| Smoker | 1.48  (0.71, 3.08) | 0.2999 | 1.807  (0.89, 3.69) | 0.1041 | 2.905  (0.97, 8.67) | 0.0561 | 2.413  (0.80, 7.25) | 0.1169 |
| Diabetes | 0.92  (0.04, 2.12) | 0.8504 | 0.620  (0.30, 1.29) | 0.2021 | 0.844  (0.35, 2.03) | 0.7039 | 0.690  (0.28, 1.68) | 0.4118 |
| Active Malignancy | 2.20  (1.09, 4.45) | 0.0277 | 1.003  (0.51,1.96) | 0.9932 | 0.875  (0.39, 1.96) | 0.7452 | 1.249  (0.51, 3.08) | 0.6283 |
| Inactive/Past Malignancy | 1.06  (0.50, 2.25) | 0.8798 | 1.016  (0.52, 2.00) | 0.9627 | 0.687  (0.31, 1.52) | 0.3531 | 0.785  (0.34, 1.82) | 0.5730 |
| Nephrotic syndrome | 4.24  (0.69, 26.1) | 0.1196 | 3.290  (0.36, 30.0) | 0.2908 | NC  NC | 0.9848 | NC  NC | 0.9857 |
| Renal Insufficiency | 8.53  (0.87, 83.8) | 0.0661 | 2.444  (0.25, 23.9) | 0.4424 | NC  NC | 0.9864 | NC  NC | 0.9805 |
| Liver Disease | 7.10  (2.09, 24.2) | 0.0017 | 4.784  (1.03, 22.2) | 0.0455 | 3.263  (0.41, 25.8) | 0.2626 | 2.762  (0.35, 21.9) | 0.3366 |
| Personal history of DVT/PE | 0.84  (0.40, 1.77) | 0.6490 | 0.878  (0.46, 1.68) | 0.6927 | 1.195  (0.53, 2.72) | 0.6707 | 0.968  (0.42, 2.23) | 0.9383 |
| Family history of DVT/PE | 0.90  (0.23, 3.44) | 0.8722 | 0.790  (0.25, 2.54) | 0.6931 | 0.490  (0.14, 1.72) | 0.2646 | 0.410  (0.12, 1.45) | 0.1654 |
| Hereditary/Acquired thrombophilia | <0.001  NC | 0.9829 | <0.001  NC | 0.9789 | 0.375  (0.06, 2.32) | 0.2917 | 0.317  (0.05, 1.97) | 0.2175 |
| Recent hospitalization (< 4 weeks) | 0.58  (0.22, 1.50) | 0.2558 | 0.662  (0.31, 1.41) | 0.2861 | 0.733  (0.30, 1.78) | 0.4925 | 0.942  (0.36, 2.49) | 0.9041 |
| Recent Surgery (< 4 weeks) | 1.66  (0.62, 4.48) | 0.3135 | 1.420  (0.54, 3.77) | 0.4819 | 0.963  (0.30, 3.07) | 0.9495 | 1.189  (0.33, 4.32) | 0.7923 |
| COVID-19 | <0.001  NC | 0.9847 | 0.261  (0.03, 0.56) | 0.2478 | 0.248  (0.03, 1.82) | 0.1704 | 0.210  (0.03, 1.54) | 0.1250 |
| Male | 1.32  (0.69, 2.53) | 0.3986 | 1.332  (0.75, 2.53) | 0.3239 | 2.016  (1.02, 4.03) | 0.0475 | 2.167  (1.04, 4.50) | 0.0379 |
| Central PE | 0.89  (0.47,1.68) | 0.7121 | 0.811  (0.46, 1.43) | 0.4680 | 1.262  (0.62, 2.57) | 0.5197 | 1.489  (0.70, 3.18) | 0.3037 |
|  | 2.31 |  | 1.02 |  | 1.55 |  | 1.88 |  |
| DVT Positive | (1.16, 4.63) | 0.0180 | (0.56, 1.86) | 0.9487 | (0.73, 3.31) | 0.2537 | (0.84, 4.19) | 0.1226 |
| RHS on CTPA | 1.80  (0.93, 3.49) | 0.0812 | 1.407  (0.78, 2.56) | 0.2613 | 1.534  (0.72, 3.26) | 0.2668 | 1.944  (0.85, 4.45) | 0.1151 |
| Any of RVD, RVH, SF/SB* | 1.42  (0.72, 2.79) | 0.3081 | 1.842  (0.97, 3.50) | 0.0624 | 1.262  (0.57, 2.78) | 0.5643 | 1.205  (0.53, 2.76) | 0.6583 |
| Race Black v. White | 0.98  (0.34, 2.88) | 0.9755 | 1.077  (0.42, 2.74) | 0.8768 | 1.612  (0.45, 5.82) | 0.4657 | 2.197  (0.48, 9.97) | 0.3079 |
| Race Hispanic v. White | 1.88  (0.68, 5.17) | 0.2231 | 2.290  (0.78, 6.73) | 0.1318 | 2.276  (0.50, 10.4) | 0.2879 | 1.953  (0.43, 8.94) | 0.3886 |
| Race Other v. White | 2.95  (0.40, 21.6) | 0.2873 | 2.643  (0.27, 26.0) | 0.4044 | 0.854  (0.09, 8.47) | 0.8925 | 0.732  (0.07, 7.28) | 0.7903 |
| BMI>28.1 | 0.84  (0.45, 1.57) | 0.5818 | 0.669  (0.338, 1.17) | 0.1601 | 1.037  (0.52, 2.06) | 0.9177 | 1.118  (0.54, 2.30) | 0.7628 |
| Age >62 years v. <62 years | 1.01  (0.99, 1.03) | 0.4141 | 1.001  (0.98, 1.02) | 0.9179 | 0.999  (0.98, 1.02) | 0.8994 | 1.002  (0.98, 1.03) | 0.8468 |
|  |  |  |  |  |  |  |  |  |

**Supplemental Table 1 Notes:**

^A^NC = could not be calculated due to zero cells in the cross tabulation.

Abbreviations: AT = antithrombin; CHF = Congestive Heart Failure; EF = (Left Ventricular) Ejection Fraction; COPD = Chronic Obstructive Pulmonary Disease; DVT = Deep Vein Thrombosis; PE = Pulmonary Embolism; RHS = Right Heart Strain; CTPA = Computed Tomography Pulmonary Angiography; RVD = Right Ventricular Dilatation; RVH = Right Ventricular Hypokinesis, SF/SB = Septal Flattening or Septal Bowing; BMI = Body Mass Index
