# Supplementary material for: The Thromboembolism Heparinization and AntithrombiN Observational Study (THANOS-1)
Source: Res Pract Thromb Haemost. 2026 Jan 27;10(1):103367. doi: 10.1016/j.rpth.2026.103367 (PMC12934317; doi:10.1016/j.rpth.2026.103367)
Supplement: Supplementary Table 2 [file mmc2.docx]

**Supplemental Table 2:** Multivariate analyses of clinical factors and hospital factor LOS associated with AT activity

|  | Covariate | Odds Ratio  (95% CI) | p-value |
| --- | --- | --- | --- |
| AT <80 v. >=80 | CHF (EF < 45%) | 2.26 (0.48, 10.57) | 0.2990 |
|  | Stroke | 0.08 (0.01, 1.01) | 0.0507 |
|  | Active malignancy | 1.84 (0.63, 5.31) | 0.2625 |
|  | Nephrotic syndrome | 3.47 (0.38, 31.28) | 0.2675 |
|  | Renal insufficiency | 12.56 (0.22, 706.2) | 0.2184 |
|  | Liver disease | 7.78 (1.58, 38.24) | 0.0115 |
|  | DVT positive | 1.50 (0.61, 3.74) | 0.3788 |
|  | RHS on CTPA | 1.12 (0.43, 2.91) | 0.8207 |
|  | Length of Stay (hours) | 1.01 (1.00, 1.01) | 0.0005 |
|  |  |  |  |
| AT <90 v. >=90 | CHF (EF < 45%) | 3.66 (0.71, 18.82) | 0.1206 |
|  | Asthma/COPD | 2.31 (0.94, 5.70) | 0.0682 |
|  | Smoker | 1.85 (0.83, 4.12) | 0.1342 |
|  | Liver disease | 3.31 (0.65, 16.93) | 0.1515 |
|  | RHS on echocardiogram | 1.60 (0.79, 3.24) | 0.1885 |
|  | Black v. White race | 1.43 (0.51, 3.95) | 0.4949 |
|  | Hispanic v. White race | 1.78 (0.54, 5.88) | 0.3410 |
|  | Other v. White race | 4.59 (0.43, 49.10) | 0.2079 |
|  | BMI | 0.79 (0.42, 1.50) | 0.4728 |
|  | Length of stay (hours) | 1.00 (1.00, 1.01) | 0.0020 |
|  |  |  |  |
| AT <100 v. >=100 | Smoker | 2.98 (0.95, 9.33) | 0.0607 |
|  | COVID-19 | 0.12 (0.01, 1.24) | 0.0752 |
|  | Gender (male v. female) | 2.13 (1.03, 4.39) | 0.0418 |
|  | Length of stay (hours) | 1.00 (1.00, 1.01) | 0.0505 |
|  |  |  |  |
| AT <110 v. >=110 | Smoker | 2.99 (0.59, 15.20) | 0.1875 |
|  | Family history of DVT/PE | 0.27 (0.06, 1.16) | 0.0774 |
|  | COVID-19 | 0.05 (0.00, 0.90) | 0.0423 |
|  | Gender (male v. female) | 2.25 (0.90, 5.63) | 0.0834 |
|  | DVT positive | 1.35 (0.54, 3.38) | 0.5225 |
|  | RHS on CTPA | 1.87 (0.69, 5.02) | 0.2161 |
|  | Length of stay (hours) | 1.00 (1.00, 1.01) | 0.1052 |

**Supplemental Table 2 Notes:**

Because ICU admission and hospital LOS are colinear, we added hospital LOS and ICU admission separately into the multivariate model with patient factors.

Abbreviations: AT = antithrombin; CHF = Congestive Heart Failure; EF = (Left Ventricular) Ejection Fraction; DVT = Deep Vein Thrombosis; RHS = Right Heart Strain; CTPA = Computed Tomography Pulmonary Angiography; BMI = Body Mass
