# Supplementary material for: The Thromboembolism Heparinization and AntithrombiN Observational Study (THANOS-1)
Source: Res Pract Thromb Haemost. 2026 Jan 27;10(1):103367. doi: 10.1016/j.rpth.2026.103367 (PMC12934317; doi:10.1016/j.rpth.2026.103367)
Supplement: Supplementary Table 3 [file mmc3.docx]

**Supplemental Table 3:** Multivariate analyses of clinical factors and hospital factor ICU Admission associated with AT activity

|  | Covariate | Odds Ratio  (95% CI) | p-value |
| --- | --- | --- | --- |
| AT <80 v. >=80 | CHF (EF < 45%) | 1.86 (0.42, 8.24) | 0.4131 |
|  | Stroke | 0.16 (0.01, 1.81) | 0.1393 |
|  | Active Malignancy | 2.29 (0.85, 6.18) | 0.1002 |
|  | Nephrotic syndrome | 1.29 (0.15, 11.09) | 0.8178 |
|  | Renal Insufficiency | 12.25 (0.49, 309.1) | 0.1283 |
|  | Liver Disease | 7.07 (1.55, 32.34) | 0.0117 |
|  | DVT positive | 2.14 (0.91, 5.01) | 0.0802 |
|  | RHS on CTPA | 1.53 (0.63, 3.73) | 0.3505 |
|  | ICU Admission | 2.05 (0.78, 5.40) | 0.1477 |
|  |  |  |  |
| AT <90 v. >=90 | CHF (EF < 45%) | 3.15 (0.61,16.23) | 0.1707 |
|  | Asthma/COPD | 2.49 (1.03, 5.97) | 0.0419 |
|  | Smoker | 1.93 (0.86, 4.31) | 0.1086 |
|  | Liver Disease | 4.72 (0.95, 23.38) | 0.0576 |
|  | Right heart strain on echo | 1.44 (0.69, 2.97) | 0.3295 |
|  | Black v. White race | 1.41 (0.52, 3.84) | 0.5052 |
|  | Hispanic v. White race | 1.63 (0.50, 5.29) | 0.4138 |
|  | Other v. White race | 4.29 (0.42, 44.32) | 0.2212 |
|  | BMI >28.1 | 0.70 (0.38, 1.31) | 0.2681 |
|  | ICU Admission | 2.42 (1.01, 5.79) | 0.0480 |
|  |  |  |  |
| AT <100 v. >=100 | Smoker | 4.27 (1.17, 15.55) | 0.0276 |
|  | COVID-19 | 0.12 (0.01, 1.27) | 0.0777 |
|  | Gender (male v. female) | 1.92 (0.93, 3.97) | 0.0781 |
|  | ICU Admission | 2.60 (0.85, 7.96) | 0.0948 |
|  |  |  |  |
| AT <110 v. >=110 | Smoker | 6.66 (0.71, 62.11) | 0.0960 |
|  | Family history of DVT/PE | 0.21 (0.05, 0.95) | 0.0422 |
|  | COVID-19 | 0.03 (0.00, 0.93) | 0.0453 |
|  | Gender (male v. female) | 1.78 (0.71, 4.47) | 0.2230 |
|  | DVT positive | 1.32 (0.52, 3.35) | 0.5614 |
|  | RHS on CTPA | 1.77 (0.65, 4.82) | 0.2663 |
|  | ICU Admission | 3.16 (0.65, 15.47) | 0.1551 |

**Supplemental Table 3 Notes:**

Because ICU admission and hospital LOS are colinear, we added hospital LOS and ICU admission separately into the multivariate model with patient factors.

Abbreviations: AT = antithrombin; CHF = Congestive Heart Failure; EF = (Left Ventricular) Ejection Fraction; DVT = Deep Vein Thrombosis; RHS = Right Heart Strain; CTPA = Computed Tomography Pulmonary Angiography; ICU = Intensive Care Unit; COPD = Chronic Obstructive Pulmonary Disease; BMI = Body Mass Index
