# Supplementary material for: The Thromboembolism Heparinization and AntithrombiN Observational Study (THANOS-1)
Source: Res Pract Thromb Haemost. 2026 Jan 27;10(1):103367. doi: 10.1016/j.rpth.2026.103367 (PMC12934317; doi:10.1016/j.rpth.2026.103367)
Supplement: Supplementary Table 4 [file mmc4.docx]

**Supplemental Table 4:** Time to Therapeutic Status by Initial Antithrombin Level

| Antithrombin Level on Day 0 | Hazard Ratio (95% CI) | p-value |
| --- | --- | --- |
| <80 vs >= 80 | 0.94 (0.66, 1.35) | 0.755 |
| <90 vs >= 90 | 1.23 (0.88, 1.73) | 0.223 |
| <100 vs >= 100 | 1.28 (0.82, 2.00) | 0.279 |
| <110 vs >= 110 | 1.44 (0.89, 2.33) | 0.142 |
| Continuous AT by 10 | 0.98 (0.91, 1.06) | 0.572 |
